# Enhanced glucose tolerance in pancreatic-derived factor (PANDER) knockout C57BL/6 mice

**DOI:** 10.1242/dmm.016402

**Published:** 2014-09-12

**Authors:** Shari L. Moak, Grace C. Dougan, Catherine B. MarElia, Whitney A. Danse, Amanda M. Fernandez, Melanie N. Kuehl, Mark G. Athanason, Brant R. Burkhardt

**Affiliations:** 1 Department of Cell Biology, Microbiology and Molecular Biology, University of South Florida, 4202 East Fowler Avenue, Tampa, FL 33620, USA.; 2 Department of Pediatrics, University of South Florida, 12901 Bruce B. Downs Boulevard MDC 62, Tampa, FL 33612, USA.

**Keywords:** Pancreatic-derived factor, FAM3B, Knockout model, Glycemic regulation, Hepatic insulin signaling, Glucose tolerance

## Abstract

Pancreatic-derived factor (PANDER; also known as FAM3B) is a uniquely structured protein strongly expressed within and secreted from the endocrine pancreas. PANDER has been hypothesized to regulate fasting and fed glucose homeostasis, hepatic lipogenesis and insulin signaling, and to serve a potential role in the onset or progression of type 2 diabetes (T2D). Despite having potentially pivotal pleiotropic roles in glycemic regulation and T2D, there has been limited generation of stable animal models for the investigation of PANDER function, and there are no models on well-established genetic murine backgrounds for T2D. Our aim was to generate an enhanced murine model to further elucidate the biological function of PANDER. Therefore, a pure-bred PANDER knockout C57BL/6 (PANKO-C57) model was created and phenotypically characterized with respect to glycemic regulation and hepatic insulin signaling. The PANKO-C57 model exhibited an enhanced metabolic phenotype, particularly with regard to enhanced glucose tolerance. Male PANKO-C57 mice displayed decreased fasting plasma insulin and C-peptide levels, whereas leptin levels were increased as compared with matched C57BL/6J wild-type mice. Despite similar peripheral insulin sensitivity between both groups, hepatic insulin signaling was significantly increased during fasting conditions, as demonstrated by increased phosphorylation of hepatic PKB/Akt and AMPK, along with mature SREBP-1 expression. Insulin stimulation of PANKO-C57 mice resulted in increased hepatic triglyceride and glycogen content as compared with wild-type C57BL/6 mice. In summary, the PANKO-C57 mouse represents a suitable model for the investigation of PANDER in multiple metabolic states and provides an additional tool to elucidate the biological function and potential role in T2D.

## INTRODUCTION

Pancreatic-derived factor (PANDER; also known as FAM3B), discovered in 2002, is a 235-amino-acid protein that belongs to the family with sequence similarity 3 (FAM3) gene family (Zhu et al., 2002). This cytokine-like gene family (based on predicted secondary structure) has four members described as FAM3A, FAM3B, FAM3C and FAM3D. FAM3B was later named PANDER because this hormone is robustly expressed in and secreted from the endocrine pancreas (α cells and β cells, specifically) and, to a lesser extent, other tissues such as the liver, small intestine and prostate (Li et al., 2011; Li et al., 2013; Zhu et al., 2002). From the ostensible recognition of folds (ORF) algorithm that was used in PANDER’s initial discovery, it was determined that PANDER (then identified as FAM3B) had a predicted typical four-helix bundle secondary structure that is present in many known cytokines (Aurora and Rose, 1998; Zhu et al., 2002). However, more recently, elucidated crystal structures of murine secreted PANDER revealed a previously unknown and unique globular β-β-α fold (Johansson et al., 2013). PANDER comprises two anti-parallel β sheets lined by three short helices arranged to form a highly conserved water-filled cavity that does not resemble any currently known cytokine, thereby contradicting the earlier predictive models (Johansson et al., 2013). This structure is conserved among other members of the FAM3 family. The homologous FAM3C, also known as interleukin-like EMT inducer (ILEI), has been previously demonstrated to be involved with the epithelial-mesenchymal transition and shows similar structure (Lahsnig et al., 2009; Song et al., 2013; Waerner et al., 2006). Therefore, PANDER and this gene family might comprise a newly identified class of signaling molecules that act in ways distinct from those of other known cytokines or hormones.

Initial *in vitro* studies regarding PANDER have demonstrated a potential role in glycemia regulation. Glucose has been shown to significantly enhance *Pander* promoter activity and secretion from β cells and pancreatic islets (Burkhardt et al., 2005; Wang et al., 2008; Yang et al., 2005). In addition, PANDER is co-secreted with insulin in response to glucose (Carnegie et al., 2010; Xu et al., 2005).

The mechanism of action and full biological effect of PANDER have yet to be fully elucidated *in vivo*, and this is the result of a lack of appropriate permanent and genomically integrated rodent models. The initial PANDER knockout (PANKO) model was generated on a mixed genetic background and demonstrated glucose intolerance in the presence of enhanced hepatic insulin sensitivity (HIS) with no observed differences in peripheral insulin sensitivity or fasting glycemic levels (Robert-Cooperman et al., 2010). Despite enhanced HIS as determined by hyperinsulinemic-euglycemic clamp (HEC) studies, no further characterization has been performed to identify the causative hepatic molecules or pathway for this finding, thereby leaving a gap in the understanding of hepatic PANDER action under both fasting and fed conditions. The targeted disruption of PANDER impairs pancreatic islet insulin secretion, as demonstrated by an inhibited glucose-induced response by PANKO islets, which was identified during islet perifusion and Ca^2+^-imaging studies. By contrast, neither endocrine pancreatic morphology nor insulin content is affected by the absence of PANDER. The lack of further hepatic molecular characterization and a robust phenotype from the mixed genetic background PANKO mouse led us to speculate that the lack of a congenic background in a pure-bred mouse model might be hindering the penetrance of the phenotype and confounding the results, particularly in relation to hepatic insulin sensitivity. Furthermore, a series of review articles has strongly suggested that PANDER could potentially be implicated in the onset or progression of type 2 diabetes (T2D) (Wang et al., 2012; Wilson et al., 2011; Yang and Guan, 2013), yet the impact of this gene within well-established models of T2D or insulin resistance has not been generated. Therefore, to further refine and define the biological function of PANDER within the context of a well-established model of T2D within a permissive genetic background, we generated and characterized the PANDER knockout mouse on a pure C57BL/6 genetic background.

**RESOURCE IMPACT**

**Background**

Pancreatic-derived factor (PANDER, FAM3B) was originally cloned in 2002 and is a member of the family with sequence similarity 3 (FAM3) gene superfamily. PANDER appears to serve a role in the regulation of glycemic levels, insulin action and hepatic lipogenesis. Overexpression of PANDER results in a phenomenon known as selective hepatic insulin resistance (SHIR), whereby insulin signaling is blunted, yet lipogenesis is increased. SHIR is a pathogenic paradoxical hallmark of type 2 diabetes (T2D), which is the most common global metabolic disorder with ever increasing rates. Despite numerous investigations eluding to PANDER as serving a potential role in the onset or progression of T2D, no stable animal models have been generated on well-established genetic backgrounds of T2D susceptibility. Therefore, there is a strong need for novel animal models on congenic backgrounds with discernible phenotypes for the investigation of PANDER function.

**Results**

In this study, the authors have generated a PANDER knockout mouse model on a pure C57BL/6 background (PANKO-C57) to promote the phenotypic penetrance of PANDER and to provide a useful tool for subsequent studies. In contrast to the previous PANDER knockout model, the PANKO-C57 model exhibited increased body weight, enhanced glucose tolerance during both fed and fasting conditions. This phenotype was more significant in PANKO-C57 male versus female mice; however, females still displayed overall trends in enhanced glucose tolerance. In addition, the fasting plasma insulin and C-peptide levels were concordantly decreased in the PANKO-C57 mouse, along with increased leptin levels. Hepatic insulin signaling was significantly increased during fasting conditions, as demonstrated by increased phosphorylation of hepatic protein kinase B (PKB/Akt) and adenosine monophosphate-activated protein kinase (AMPK), along with mature sterol regulatory element-binding protein 1 (SREBP-1) expression in the PANKO-C57 model. Insulin stimulation of PANKO-C57 mice resulted in increased hepatic triglyceride and glycogen content when compared with C57BL/6J wild-type mice.

**Implications and future directions**

Altogether, the PANDER mouse model has enhanced our understanding of the mechanistic implications of PANDER function and has unified many of the prior conflicting findings regarding its role in glycemic regulation. The study provides evidence that this generated PANDER knockout animal model, derived on a well-established background for T2D, displays an enhanced phenotype that can be easily employed in future investigations. Given the limited availability of current PANDER animal models, the PANKO-C57 mouse suitably fills this important gap. The PANKO-C57 model has a strong breeding capacity and a discernible phenotype to allow for the future creation of additional animal models and for investigations to evaluate the role of PANDER in both T2D and glycemic regulation.

## RESULTS

### Decreased fasting glycemia in PANKO-C57 mice

**Fig. 1.**
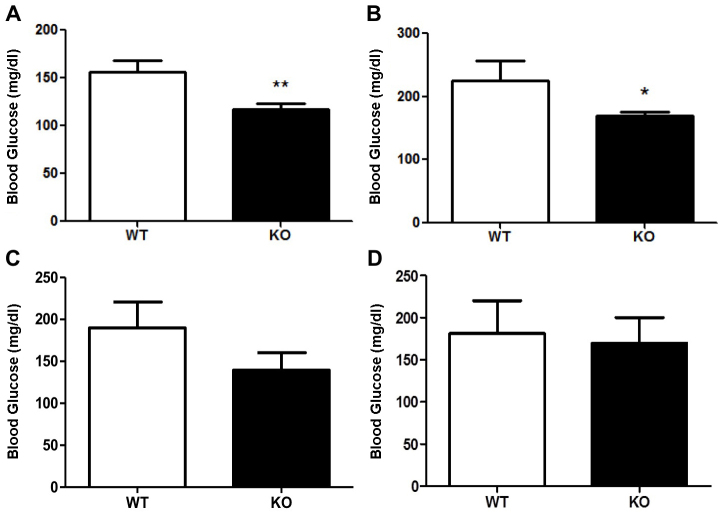
**Long- and short-term fasting glycemic measurements of PANKO-C57 mice.** Mice of both genders were 4 months of age. (A) Long-term fasting (overnight, 16 hours) blood glucose measurements of male PANKO-C57 (KO) and WT mice (*n*=12–15). (B) Short-term fasting (4 hours) blood glucose measurements of males (*n*=8–11). Values are expressed as means±s.e.m. (C) Long-term fasting blood glucose measurements of female PANKO-C57 and WT mice (*n*=7–12). (D) Short-term fasting (4 hours) blood glucose measurements of females (*n*=6–10). *P<0.05, **P<0.01, Student’s *t*-test.

Before experimentation, all mice were genotyped as a confirmatory measure following receipt of PANKO-C57 mice from Jackson Laboratories using primers described in Materials and Methods (data not shown). The effect of PANDER on fasting glycemic levels was determined by measuring blood glucose following a short-(4 hours) and long-term (16 hours) fast. PANKO-C57 male mice aged 4 months displayed significantly decreased blood glucose levels after a long-term fast compared with age- and sex-matched C57BL/6 mice (*P*<0.01) (Fig. 1A). Decreased fasting glycemia levels were also observed following a short-term fast, with blood glucose values of 169.3 mg/dl±6.9 (±s.e.m.) vs. 225.8 mg/dl±30.5 (*P*<0.05) (Fig. 1B). A similar but non-statistical trend was observed with female PANKO-C57 long- and short-term fasting mice (Fig. 1C,D). In summary, the absence of PANDER promoted decreased fasting glycemic levels that had not been previously observed or reported in the mixed genetic background PANDER knockout or adenovirally delivered PANDER siRNA models, revealing a discernible phenotype within this background (Li et al., 2011; Robert-Cooperman et al., 2010).

### Enhanced glucose tolerance in PANKO-C57 mice

**Fig. 2.**
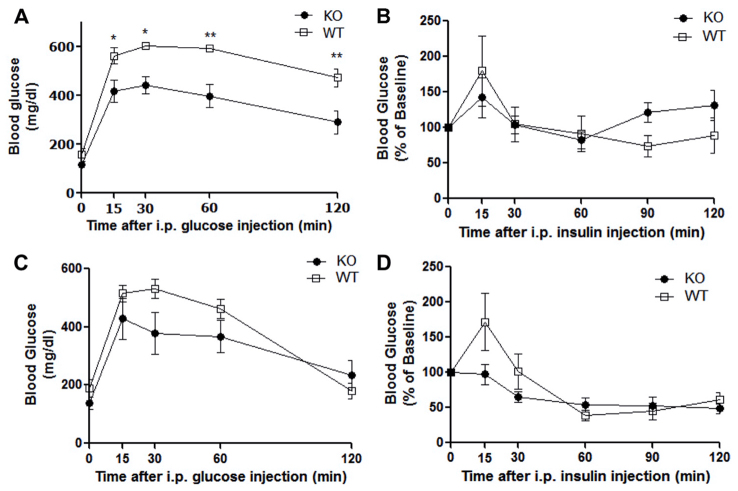
**Enhanced glucose tolerance in male PANKO-C57 mice.** All mice were 4 months of age. (A) Intraperitoneal (i.p.) glucose tolerance tests (GTTs) were performed following overnight fast (~16 hours) on male PANKO-C57 (KO) and WT mice through injection of glucose at 2 g/kg of body weight and measurement of plasma glucose concentration at the indicated time points (*n*=8–12). (B) Intraperitoneal insulin tolerance tests (ITTs) were performed on male PANKO-C57 and WT mice following a 4-hour fast by injection of insulin at 1 unit/kg of body weight and subsequent measurement of plasma glucose concentration at all indicated time points. Results are presented as the percentage of the baseline glucose concentration measured at time point 0 (*n*=8–11). (C) GTTs performed on female PANKO-C57 and WT mice as described above (*n*=7–12). (D) ITTs performed on female PANKO-C57 and WT mice as described above (*n*=7–11). Values are expressed as the mean±s.e.m. *P<0.05, **P<0.01 as determined by using two-way ANOVA.

To evaluate the effect of PANDER during postprandial conditions in our model, PANKO-C57 and wild-type (WT) mice of both genders were examined by using glucose tolerance tests (GTTs). GTTs performed at 4 months of age revealed enhanced glucose tolerance in male PANKO-C57 mice based on decreased glycemic levels throughout the duration of the GTT (Fig. 2A). Significantly lower blood glucose measurements were observed in male PANKO-C57 mice compared with WT mice during the course of the GTT (two-way ANOVA, *P*<0.05). Insulin tolerance tests (ITTs) were performed to examine peripheral insulin sensitivity. Responses to intraperitoneal-injected insulin were shown to be similar between PANKO-C57 and WT mice (Fig. 2B). With regard to female PANKO-C57 mice, a similar trend of enhanced glucose tolerance was measured during the GTT but without statistical significance (Fig. 2C). Insulin sensitivity during the ITT was similar between female PANKO-C57 and WT mice (Fig. 2D). In summary, the absence of PANDER promoted enhanced glucose tolerance without significantly affecting peripheral insulin sensitivity in a male dominant fashion. This was not observed in our prior PANKO mixed genetic background model and demonstrates an enhanced metabolic phenotype in the pure C57BL/6 model (Robert-Cooperman et al., 2010).

### PANKO-C57 male and female mice have increased body weight

**Fig. 3.**
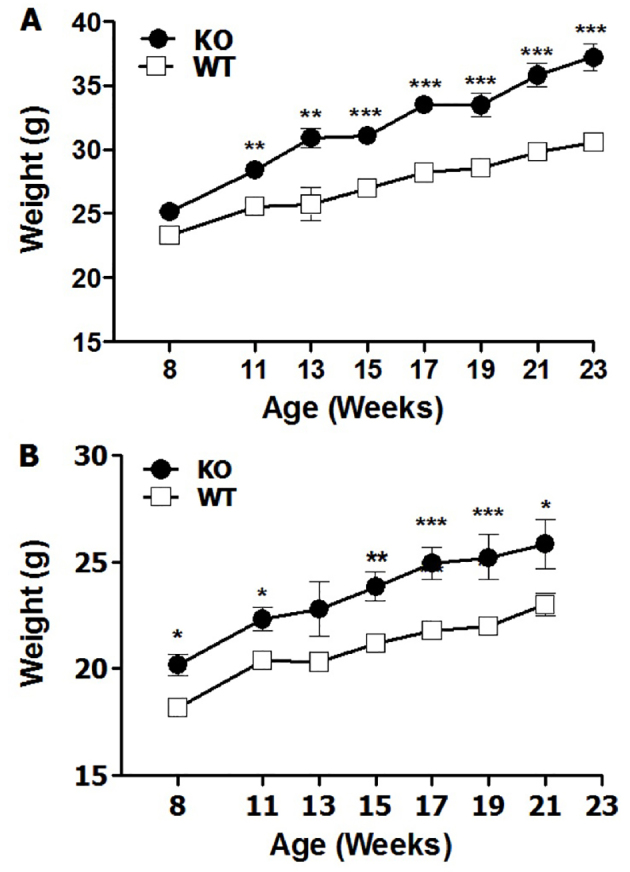
**Increased body weight of male and female PANKO-C57 mice.** Body weight measurements were recorded at similar times from 8 to 23 weeks of age following an overnight fast. (A) Male PANKO-C57 (KO) and WT mice weight over time (*n*=18–27). (B) Female PANKO-C57 and WT mice weight over time (*n*=13–22). Values are expressed as the means±s.e.m. *P<0.05, **P<0.01, ***P<0.001 as determined by using two-way ANOVA.

To further characterize the PANKO-C57 mice, the longitudinal body weight was evaluated from 8 to 23 weeks of age. Beginning at 8 weeks of age upon feeding with normal chow *ad libitum*, PANKO-C57 male mice presented with significantly increased weights compared with age- and gender-matched WT mice [up to 23 weeks of age (Fig. 3A)]. Measurements of body weight were terminated at 23 weeks of age. This same trend was observed in female mice, and although significance diminished over time, PANKO-C57 female mice remained significantly heavier than WT mice at 21 weeks of age (Fig. 3B). Increased body weight has not been reported or observed in previous animal models and reveals a potential previously overlooked function of PANDER in the area of whole-body homeostasis. Furthermore, the appearance of this phenotype in both genders supports the distinction of this model from other PANDER animal models (Li et al., 2011; Robert-Cooperman et al., 2010; Wilson et al., 2010).

### Fasting hormone levels in PANKO-C57 mice

**Fig. 4.**
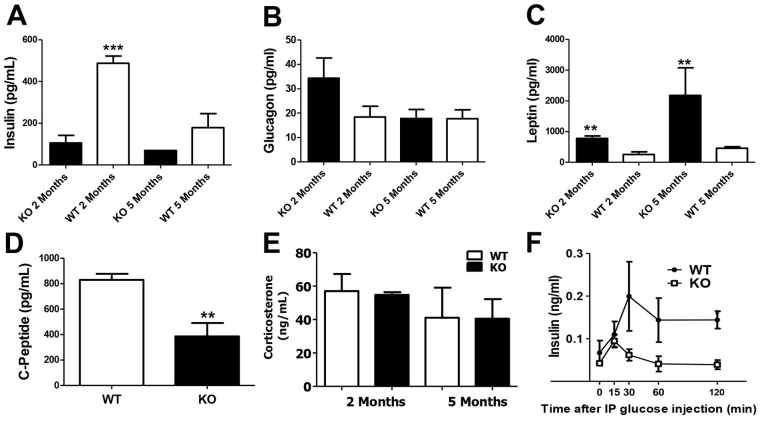
**Hormonal evaluation of male PANKO-C57 during fasting.** The plasma levels of (A) insulin, (B) glucagon and (C) leptin were measured during fasting at 2 and 5 months of age in male PANKO-C57 (KO) and WT mice using the Mouse Metabolic Hormone Panel (Millipore) as measured by the MAGPIX^®^ Luminex system (*n*=4–6). (D) Overnight fasting levels of C-peptide from mice aged 2 months using the MAGPIX^®^ Luminex system as above (*n*=4). (E) Corticosterone levels were measured from plasma collected during fasting conditions at 2 and 5 months of age using the Corticosterone EIA Kit (Enzo Life Sciences) (*n*=3). (F) Plasma insulin levels during the course of GTTs (*n*=3–5). Values are expressed as means±s.e.m. *P<0.05, **P<0.01, ***P<0.001 as determined by using Student’s *t*-test for A–E and two-way ANOVA in F. IP, intraperitoneal.

Following the combined gender phenotyping, we focused our study on PANKO-C57 males in order to identify whether hormonal differences accounted for the observed decreased glycemic levels during fasting in the male PANKO-C57 mice. Therefore, the plasma levels of insulin, C-peptide, glucagon, leptin and corticosterone during fasting were measured at 2 and 5 months of age in male mice (Fig. 4). At 2 months of age, PANKO-C57 male mice presented with significantly reduced insulin levels (Fig. 4A). Glucagon levels were similar between PANKO-C57 and WT mice at 2 and 5 months of age (Fig. 4B). Interestingly, leptin levels were increased in PANKO-C57 mice in both age groups (Fig. 4C). C-peptide levels at 2 months of age were significantly decreased and concordant with the decreased measured insulin levels (Fig. 4D). Fasting corticosterone levels were similar between PANKO-C57 and WT mice in both age groups (Fig. 4E). In general, the hormonal results from plasma during fasting revealed characteristics of the PANKO-C57 model which had not been observed in prior investigations, such as the decreased insulin levels in 2-month-old mice and increased leptin levels (Li et al., 2011; Robert-Cooperman et al., 2010). In addition, glucose-stimulated insulin levels were decreased in the PANKO-57 mice with statistical significance at the conclusion of the glucose tolerance test (Fig. 4F). This result is consistent with previous reports indicating that the absence of PANDER can impair glucose-stimulated insulin secretion (Robert-Cooperman et al., 2010; Robert-Cooperman et al., 2011).

### Enhanced insulin-stimulated hepatic glycogen and triglyceride content

**Fig. 5.**
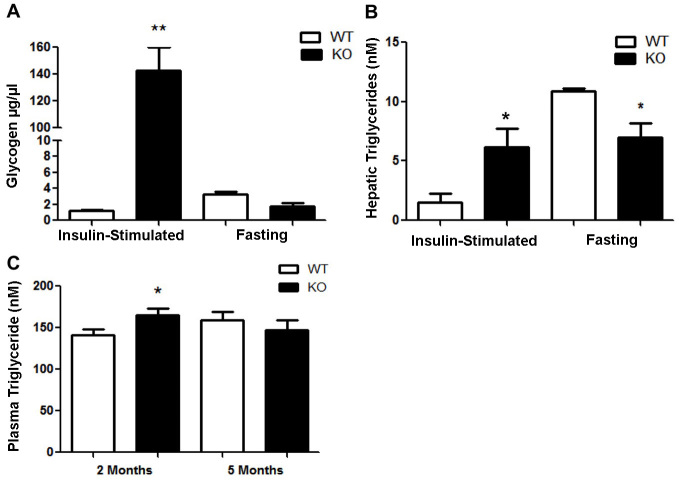
**Hepatic glycogen, triglyceride and gluconeogenic content and expression in PANKO-C57.** (A) Hepatic glycogen content (in μg/μl of tissue lysate) measurement of male PANKO-C57 (KO) and WT mice at 4–5 months of age following insulin stimulation (2 U/kg of body weight) or an overnight fast using the Glycogen Assay Kit (Abcam^®^) (*n*=3 per group). (B) Hepatic triglyceride content evaluation of 4-to 5-month-old male PANKO-C57 and WT mice as described in A, using 100 mg of liver tissue and the Triglyceride Quantification Kit (Abcam^®^) (*n*=3). (C) Serum triglycerides from serum after fasting of PANKO-C57 and WT male mice were analyzed longitudinally at 2 and 5 months of age using the Triglyceride Quantification Kit (Abcam^®^) (*n*=5). Values are expressed as the mean+s.e.m. *P<0.05, **P<0.01 as determined by using Student’s *t*-test.

To elucidate further downstream metabolic consequences of PANDER loss, we examined hepatic glycogen and triglyceride levels following insulin-stimulated and fasting conditions in male mice. In terms of hepatic glycogen content, 15 minutes after stimulation with 2 units of insulin per kg of body weight, the glycogen concentration within PANKO-C57 mouse livers was significantly increased, over 120-fold, compared with that of WT mice (Fig. 5A). Hepatic glycogen levels were not significantly different following conditions of fasting in the PANKO-C57 model when compared with those of WT controls (Fig. 5A). The hepatic triglyceride concentration was also increased in PANKO-C57 mice during insulin-stimulated conditions as compared with that of WT mice (Fig. 5B). During fasting conditions, hepatic triglyceride concentration was significantly decreased in PANKO-C57 mice as compared to that of WT counterparts (Fig. 5B). The circulating plasma triglyceride concentration was measured after an overnight fast and was significantly increased in 2-month-old PANKO mice as compared with WT mice, but not at 5 months of age (Fig. 5C). In summary, the liver responds very well to insulin in the PANKO-C57 model when it is stimulated for the production of glycogen, but not when it is stimulated to produce triglycerides. In addition, the lowered fasting glucose levels suggest that hepatic gluconeogenesis is effectively suppressed by insulin.

### PANKO-C57 male mice display enhanced hepatic signaling

**Fig. 6.**
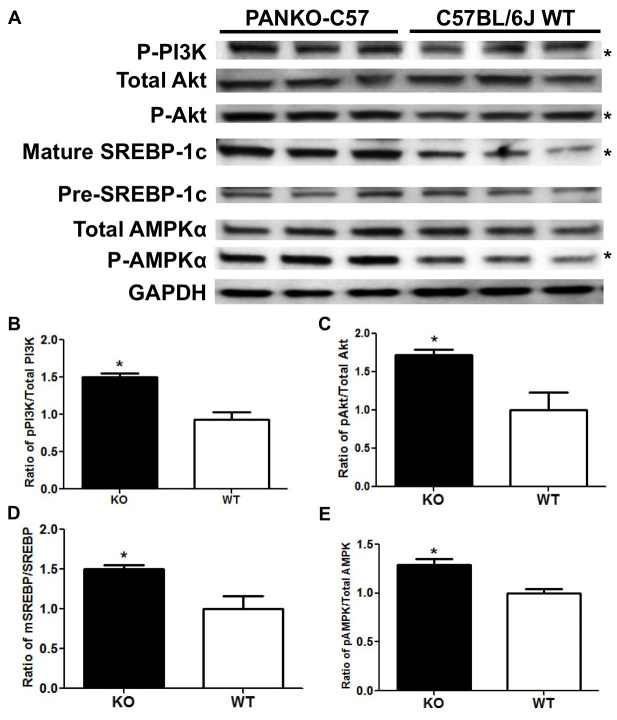
**Western blot analysis of PANKO-C57 hepatic signaling.** (A) Western blot analysis for the protein levels of phosphorylated PI3K (p-PI3K; at residue Tyr 508), total and phosphorylated Akt (p-Akt; at residue Thr 308), mature and precursor (pre) SREBP-1, total and phosphorylated AMPKα (p-AMPKα; at residue Thr 172) in lysates from the livers of male PANKO-C57 and WT mice that had been subject to overnight fast. GAPDH served as loading control. The first three lanes correspond to PANKO-C57 mice and the final three lanes correspond to age- and gender-matched C57BL/6J WT mice. *Statistical significance of *P*<0.05 as determined by measuring using ImageJ as detailed in B–E. (B) Densitometry analysis of hepatic protein levels of phosphorylated PI3K, (C) Akt, (E) AMPK and (D) levels of mature SREBP-1 (mSREBP-1). The levels were determined and normalized to the total protein levels of the respective protein followed by normalization of the total protein to GAPDH (*n*=3). Values are expressed as the mean+s.e.m. *P<0.05 as determined by using unpaired Student’s *t*-test.

Previous investigations by our laboratory and others have provided evidence that the liver is a major target organ of PANDER (Li et al., 2011; Robert-Cooperman et al., 2010; Robert-Cooperman et al., 2014; Wilson et al., 2010). In addition, HEC studies that were performed in the former PANKO mouse model on a mixed genetic background indicated enhanced hepatic insulin sensitivity, but the mechanism and crucial signaling molecules accounting for this were not characterized or identified (Robert-Cooperman et al., 2010). Therefore, we evaluated the effect of PANDER on crucial hepatic signaling molecules that are involved in canonical insulin signaling and triglyceride synthesis within the PANKO-C57 model. Western blot analysis of numerous hepatic insulin signaling molecules was performed on extracts from the livers of male PANKO-C57 and WT mice that were excised after an overnight fast or insulin stimulation (Fig. 6).

During fasting conditions, increased levels of phosphorylated hepatic signaling molecules were observed in the PANKO-C57 model (Fig. 6). Significantly increased phosphorylated levels of phosphatidylinositol 3-kinase and (PI3K), protein kinase B (PKB/Akt) and adenosine monophosphate-activated protein kinase (AMPK) were observed during fasting conditions (Fig. 6A–C). Furthermore, significantly increased levels of mature sterol regulatory element-binding protein 1 (SREBP-1) were also observed and could provide a mechanistic explanation for increased triglyceride production (Fig. 6D). During insulin stimulatory conditions, increased levels phosphorylated PI3K, Akt and AMPK were observed in the livers of PANKO-C57 mice as compared to those of WT controls (data not shown). However, following densitometry analysis, differences were not statistically significant (data not shown). Taken together, hepatic signaling in the PANKO-C57 model shows an overall enhancement of essential insulin signaling, particularly in the fasted state, which is a unique observation within the PANKO-C57 model and suggests that PANDER serves a role in the regulation of hepatic signaling during both feeding and fasting conditions.

## DISCUSSION

### Distinct metabolic differences observed in the PANKO-C57 model as compared to those on a mixed genetic background

In contrast to the previous mixed PANKO model, the PANKO-C57 mice demonstrated (1) enhanced glucose tolerance during GTTs and fasting conditions, (2) decreased fasting insulin and C-peptide levels, (3) increased fasting leptin levels, (4) increased body weight and (5) increased phosphorylated Akt, phosphorylated AMPK and mature SREBP-1 levels. Our original characterization of the PANDER knockout was performed on a mixed genetic background of C57BL/6 and 129Sv mice. The previous PANKO model exhibited the following characteristics as compared with WT controls, (1) glucose intolerance during GTTs but not fasting conditions, (2) both impaired insulin secretion and clearance, (3) no differences in fasting glucagon, insulin or leptin, (4) similar peripheral insulin sensitivity, (5) enhanced hepatic insulin sensitivity, and (6) no differences in body weight (Robert-Cooperman et al., 2010). Compared with the previous PANKO model, the PANKO-C57 mouse displays a much more exaggerated phenotype, particularly during fasting conditions, in terms of additional differences from WT controls. Also, the PANDER transgenic (PANTG; discussed in further detail later) and mixed PANKO models revealed distinct differences within males only, whereas, the PANKO-C57 mouse exhibited increased weight gain and matched observed metabolic differences primarily seen in females, providing further evidence of a more pronounced penetrance of phenotype on the C57BL/6 background. The influence of genetic strain on phenotype has been well documented in mice (Berglund et al., 2008; Goren et al., 2004). This difference might be attributed to the effect that the host genome can have on numerous metabolic factors, such as insulin secretion or peripheral and hepatic sensitivity (Doetschman, 2009; Kahle et al., 2013), particularly in the C57BL/6J mouse model, which serves as a common in-bred strain of diet-induced T2D (Freeman et al., 2006; Toye et al., 2005). The findings from the PANKO-C57 model were both consistent with and contrasting from those of the mixed genetic background. The primary consistent phenotype from both models was with regard to hepatic insulin signaling. Although increased hepatic insulin sensitivity was demonstrated in the mixed PANKO model, there was no reported characterization of crucial signaling molecules, such as Akt or AMPK. However, consistent with the observed hepatic insulin sensitivity reported in the prior model, the PANKO-C57 model demonstrated an enhancement of both hepatic Akt and AMPK phosphorylation in addition to other downstream effectors, such as SREBP-1. Furthermore, another concordant observation was that peripheral insulin sensitivity was unaffected in both knockout models. Taken together, the overall hypothesis that PANDER impacts glycemic levels, in part, through interaction with the liver and hepatic signaling was certainly supported. Also, the PANTG and mixed PANKO models revealed distinct differences within males only, whereas the PANKO-C57 mice exhibited increased weight gain and matched observed metabolic differences between both males and females, providing further evidence of a more pronounced penetrance of phenotype on the C57BL/6J background. The previous PANKO mixed model did show impaired insulin secretion, resulting in glucose intolerance. This matched observation was also supported somewhat in the PANKO-C57 model, as decreased fasting levels of insulin and C-peptide were reported, but this did not have any impact on glucose intolerance as previously reported in the mixed model. It is reasonable to speculate that the impaired insulin secretion is adequately compensated by enhanced hepatic insulin sensitivity and therefore does not result in an observable impaired glucose response.

### PANKO-C57 model vs. PANDER transgenic and acute models

During the preparation of this manuscript, we recently published the findings from our PANTG model (Robert-Cooperman et al., 2014). This transgenic mouse exclusively overexpresses PANDER from the pancreatic β-cell which results in an approximate 4-fold increase in circulating PANDER levels in both the fasted and fed state. The phenotype of the PANTG mouse as compared to the PANKO-C57 mouse is strikingly concordant in terms of the following observations: (1) fasting glucose, C-peptide and insulin levels are elevated (decreased in PANKO-C57), (2) hepatic insulin sensitivity is decreased, (3) hepatic phosphorylated AMPK is decreased during insulin stimulatory conditions, and (4) glucose intolerance is observed during GTTs (Robert-Cooperman et al., 2014). In general, the consistency between the observed metabolic phenotypes of the PANKO-C57 and PANTG mice strongly implicates a pleiotropic role for PANDER in the disruption of hepatic insulin signaling and the effects on glycemic levels in both the fasted and fed state. Acute models of PANDER employing transient overexpression within the liver through adenoviral delivery (Ad-PANDER) have also demonstrated concordant data (Li et al., 2011; Wilson et al., 2010). Li et al. report that overexpression of hepatic PANDER results in suppressed Akt activation, and hepatic silencing of PANDER decreased insulin resistance in *db/db* mice; however, these reports did not reveal any significant differences in glycemic levels during fasting or fed conditions (Li et al., 2011). Wilson et al. reveal that Ad-PANDER mice demonstrate increased fasting glucose and insulin levels, along with glucose intolerance (Wilson et al., 2010). However, Wilson et al. conclude that the glucose intolerance is due to elevated basal fasting glycemic levels rather than impaired insulin secretion or sensitivity. This effect has been attributed to PANDER amplifying glucagon signaling through hepatic cAMP and cAMP-response element-binding (CREB) protein, in contrast to the impaired Akt signaling that has been reported by Li et al. The observable and discernible phenotype within the PANKO-C57 model with regard to both fasting and fed glucose intolerance, which has not been seen in previous models, provides a measurable outcome and suitable metabolic end point to further investigate PANDER, and enables defined outcomes using this model that have not been observed in prior studies.

### Absence of PANDER results in increased leptin and hepatic insulin sensitivity

Another interesting finding observed uniquely in the PANKO-C57 model, is the increased serum leptin levels. Leptin is produced from adipocytes and serves a crucial role in both weight gain and appetite (Ahima and Flier, 2000). Gross differences in food consumption were not observed in the PANKO-C57 model (S.L.M., personal observation). Administration of leptin to *ob/ob* or C57BL/6J mice demonstrates improved hepatic insulin sensitivity and decreased fasting glycemia and body weight (Halaas et al., 1995; Pelleymounter et al., 1995). Circulating leptin levels are associated with increased body weight in both rodents and humans (Considine et al., 1996; Frederich et al., 1995). In addition, leptin has been demonstrated to enhance the suppression of crucial gluconeogenic enzymes, such as PEPCK. The PANKO-C57 model was similar, in most characteristics, to leptin models in terms of decreased fasting glycemia and enhanced hepatic signaling. The PANKO-C57 model demonstrated increased leptin levels and weight gain in the presence of decreased fasting and glucose-stimulated insulin levels. This result might be explained in part by leptin promoting hepatic insulin sensitivity and reducing the demand on insulin secretion by pancreatic β cells. In addition, PANDER has been shown to facilitate insulin secretion and impair hepatic insulin sensitivity. The synergistic impact of these effects could result in a pronounced insulin-sensitive state in the presence of impaired pancreatic β cell function, reducing overall circulating insulin levels. Further experimentation is needed to determine whether PANDER has a potential suppressive effect on leptin production.

### The potential impact of PANDER on type 2 diabetes

With accumulating investigations and review articles suggesting PANDER influences T2D and nonalcoholic fatty liver disease by manner of selective hepatic insulin resistance (Wang et al., 2012; Wilson et al., 2011; Yang and Guan, 2013). The majority of previous reports from both the stable and transient PANDER models suggest that PANDER disrupts insulin signaling, particularly through Akt, yet hepatic lipogenesis is increased (Li et al., 2011; Robert-Cooperman et al., 2014). Strong evidence has demonstrated that the PI3K and Akt pathway activates the sterol-regulatory element-binding proteins (SREBPs), which are considered master transcriptional regulators of lipid metabolism (Krycer et al., 2010). Therefore, PANDER-induced disruption of hepatic signaling still does not alter hepatic triglyceride production and demonstrates that PANDER can result in a selective hepatic insulin-resistant state whereby suppressed Akt signaling does not impair lipogenesis but yet still fails to inhibit gluconeogenesis. Selective hepatic insulin resistance (SHIR) is a major pathological aspect of T2D and is responsible for both hyperglycemia and dyslipidemia (Biddinger et al., 2008; Brown and Goldstein, 2008). The hepatic cellular mechanism by which PANDER induces SHIR is largely unknown but has been suggested to be either mediated through inhibited phosphorylation of AMPK or through Forkhead box 1 (FOXO1). The findings in the PANKO-C57 model certainly support the impact on hepatic signaling and have revealed not only increased levels of phosphorylated Akt but also of downstream molecules, such as mature SREBP-1, with concordant increases in hepatic triglyceride content. Therefore, both the PANKO and PANTG models reveal a similar phenotype, but this may be reconciled through speculation that the absence of PANDER promotes an overall insulin-sensitive state that stimulates both glycogen and triglyceride production. Indeed, HEC studies in the prior PANKO model have revealed decreased gluconeogenic output (Robert-Cooperman et al., 2010). HEC studies performed on the PANKO-C57 model would have provided useful data but were not conducted due to current technical limitations in performing these experiments.

### Utility of the PANKO-C57 mouse in the investigation of PANDER function

Overall, the PANKO-C57 mouse provides an excellent model for the investigation of PANDER and has revealed additional and novel findings that further elucidate the metabolic function of PANDER and role of this uniquely structured molecule in glycemic regulation and hepatic insulin signaling. Identification of the PANDER receptor would be highly valuable in the understanding of PANDER biology, along with the measurement of circulating PANDER levels during various metabolic and pathophysiological conditions, particularly those found in T2D. This study evaluating the PANKO-C57 model in combination with the investigations of others strongly alludes to PANDER being a potential therapeutic candidate for the treatment of hepatic insulin resistance and steatosis typically associated with T2D.

## MATERIALS AND METHODS

### Generation of PANDER knockout C57BL/6 (PANKO-C57) mice

The initial description of the targeted disruption of the *Pander* (*Fam3b*) gene and generation of the knockout has been previously described and performed at the Children’s Hospital of Philadelphia Research Institute (CHOP) through replacement of the transcriptional start site and first two exons of the *Pander* gene with neomycin (Robert-Cooperman et al., 2010). The PANDER knockout mouse was then backcrossed with C57BL/6 mice for seven generations at CHOP, followed by subsequent additional crossings with C57BL/6J mice following commercial donation, as described next. This strain (PANKO-C57) was subsequently donated to Jackson Laboratories (Bar Harbor, ME) and is commercially available under the strain name B6.129S6-Fam3b^tm1Bkht^/J (stock number 013788). Homozygote PANKO-C57 breeding pairs were obtained from Jackson Laboratory and shipped to the Moffitt Cancer Center’s Stabile Vivarium (Tampa, FL) for murine colony generation. All mice were fed Purina normal chow and water *ad libitum*. All PANDER knockout and C57BL/6J WT offspring were screened by using PCR amplification of genomic DNA isolated from tail tissue (DNeasy Kit, Qiagen, Germantown, MD) for knockout of the gene or for identification as WT, products of 500 and 800 bp, respectively. PCR genotype confirmation cycling conditions and primers (forward primer located in the *Pander* promoter region: 5′-CTTGTGATGGTGGATGCCCAGTT-3′ and reverse primer located within neomycin gene: 5′-CTTCCTCGTGCTTTACGGTATC-3′) were used as previously described (Robert-Cooperman et al., 2010). Primarily, confirmed PANDER knockout and C57BL/6J WT mice at 8 weeks to 6 months of age were evaluated for this study. All murine handling and experimentation adhered to protocols approved by the Institutional Animal Care and Use Committee at the University of South Florida and Moffitt Cancer Center.

### Weight measurements

PANKO-C57 and WT mice were weighed (Beckman Digital Scale) at the same time in the morning (~9 am) during *ad libitum* chow conditions in 2-to 3-week increments beginning at 8 weeks of age for both genders. Weight was measured in grams and evaluated longitudinally between both groups.

### Glucose tolerance testing and insulin measurements

GTTs were performed on mice at 16 weeks of age as previously described (Robert-Cooperman et al., 2010). In short, PANKO-C57 and WT mice were subjected to an overnight fast (>16 hours) in a cage with bedding removed ~16 hours before the GTT. Mice were then injected intraperitoneally with 2 g of glucose (Fisher Scientific) per kg of body weight. Blood glucose was measured at 0 minutes before glucose injection and at 15, 30, 60 and 120 minutes thereafter using a TRUEtrack^®^ glucometer (Nipro Diagnostics, Fort Lauderdale, FL) through collection of blood through a tail vein. Insulin levels were measured using a commercially available murine insulin ELISA kit (ALPCO Diagnostics, Salem, NH) during the glucose challenge.

### Insulin tolerance testing

ITTs were performed within a week after GTTs were performed on mice at 16 weeks of age, within a week of performing GTTs, as previously described (Robert-Cooperman et al., 2010). In brief, PANKO-C57 and WT mice were fasted for 4 hours prior to the ITT. Mice were subsequently injected with NovoLog^®^ insulin (Novo Nordisk, Plainsboro, NJ) at a concentration of 1 U/kg of body weight. Blood glucose was measured immediately before injection of insulin (0 minutes) and thereafter at 15, 30, 60, 90 and 120 minutes, as described above for GTT.

### Measurement of fasting glycemia

Mice were fasted either long term (overnight, ~16 hours) or short term (4 hours) before metabolic testing as described above. Blood glucose was measured at the same time for all mice through collection from a tail vein, as previously described. Blood glucose was averaged for each group and compared based on fasting duration (*n*=8–15).

### Immunoblotting

Western blot analysis was performed on flash-frozen livers isolated from PANKO-C57 and WT mice at 5 to 6 months of age. Mice were either fasted overnight (~16 hours) or for a short period (4 hours). For insulin stimulation experiments, mice were fasted short term and then intraperitoneally injected with insulin (NovoLog^®^, Novo Nordisk, Plainsboro, NJ) at 2 U/kg of body weight. Protein was isolated from hepatic tissue using Tissue Protein Extracting Reagent (TPER) (Thermo Fisher Scientific, Rockford, IL) and quantified using Pierce BCA Protein Assay following the manufacturer’s protocol (Thermo Fisher Scientific). Hepatic lysate (20–50 μg) was analyzed using SDS-PAGE (Pre-Cast 10% Mini-PROTEAN^®^ TGX™ gels, Bio-Rad, Hercules, CA) and electrotransferred to polyvinylidine fluoride (PVDF) membrane using iBlot semi-dry transfer apparatus (Invitrogen, Carlsbad, CA). Western blots were then probed with antibodies to detect the levels of phosphorylated PI3K at Tyr 508 (cat. no. sc-12929, Santa Cruz Biotechnologies, Santa Cruz, CA), total and phosphorylated (residue Thr 308) Akt/PKB (cat. nos. 4691 and 4056, respectively, Cell Signaling, Danvers, MA), full-length mature and precursor SREBP-1 (cat. no. sc-8984, Santa Cruz Biotechnologies) and total and phosphorylated (residue Thr 172) AMPK subunit α (AMPKα) (cat. nos. 2603 and 2535, respectively, Cell Signaling). All primary antibodies were diluted 1:1000 in StartBlock™ Block Buffer (Thermo Fisher Scientific). Protein detection was achieved using horseradish-peroxidase-conjugated goat-anti-rabbit secondary antibody (Bio-Rad) at 1:3000 dilution in StartBlock™ followed by chemiluminescence detection using Pierce ECL Western Blotting Substrate (Thermo Scientific). Protein signals were visualized using the LAS 3000 Intelligent Dark Box (Fujifilm, Stamford, CT), and relative protein levels were normalized to glyceraldehyde 3-phosphate dehydrogenase (GAPDH) loading control (cat. no. 2118, Cell Signaling). Protein expression levels were quantified and compared using ImageJ (version 1.46) densitometry analysis.

### Plasma collection for corticosterone and triglyceride assays, and endocrine hormone panel

PANKO-C57 and WT mice were subjected to fasting overnight before plasma collection. Whole blood from mice that had been subjected to fasting was obtained through submandibular vein puncture and free flow collection into BD MicroContainer^®^ (Becton, Dickinson and Company, Franklin Lakes, NJ). Whole-blood samples were immediately placed on ice before being centrifuged for 5 minutes at 13,200 r.p.m. in an Eppendorf Microfuge 5430 for plasma separation into a new Eppendorf (Eppendorf, Hauppague, NY) collection tube. The endocrine hormone panel was performed using the commercially available Milliplex Mouse Metabolic Hormone Magnetic Bead Panel MMHMAG-44K (EMD Millipore Corporation, Billerica, MA) and Luminex Multiplex Platform (MAGPIX^®^). All procedures regarding the metabolic hormone panel adhered to the manufacturer’s protocol included in the assay kit. Corticosterone EIA kit (Enzo Life Sciences, Farmingdale, NY) and Triglyceride Quantification Kit (Abcam^®^, Cambridge, MA) were utilized for the determination of corticosterone and plasma triglyceride concentrations, respectively, and both were performed in accordance with the manufacturers’ suggested protocols.

### Hepatic glycogen assay

PANKO-C57 and WT mice were either fasted (overnight) or stimulated with insulin (Novolog^®^) at a concentration of 2 U/kg of body weight prior to liver isolation. Livers were harvested and immediately flash frozen in liquid nitrogen for storage at −80°C. The Glycogen Assay Kit (ab65620) (Abcam^®^) was used according to the manufacturer’s suggested protocol, and 10 mg of liver tissue was used.

### Hepatic triglyceride assay

Overnight fasting and insulin-stimulated hepatic triglyceride content was evaluated in male PANKO and WT mice at 4 to 5 months of age. Liver tissue harvest was conducted as described above from mice that had been subjected to fasting or insulin stimulation, and the Triglyceride Quantification Kit (Abcam^®^) colorimetric assay was conducted according to the manufacturer’s protocol.

### Statistical analysis

Data are presented as mean±s.e.m. unless otherwise specified. All statistical analyses were completed using GraphPad Prism (version 5) where statistical significance between groups was determined by unpaired Student’s *t*-test or two-way ANOVA. Any *P*-value that was less than 0.05 was considered to be statistically significant.
